# Data article on disposition towards enhancing SMEs’ performance through entrepreneurial orientations: Perspectives from a developing economy

**DOI:** 10.1016/j.dib.2018.03.135

**Published:** 2018-04-03

**Authors:** Ayodotun Stephen Ibidunni, Oyebisi Mary Ibidunni, Maxwell Ayodele Olokundun, Hezekiah Olubusayo Falola, Odunayo Paul Salau, Taiye Tairat Borishade

**Affiliations:** a Department of Business Management, Covenant University, Ota, Ogun State, Nigeria; b Department of Accounting, Bells University of Technology, Ota, Ogun State, Nigeria

**Keywords:** Entrepreneurial orientation, SME performance, SMEs, Developing economy, Nigeria

## Abstract

*This article present data on the disposition of SME operators towards enhancing SMEs Performance through entrepreneurial orientations. Copies of structured questionnaire were administered to* 102 SME owners/managers. Using descriptive and standard multiple regression statistical analysis, the data described how proactiveness, risk-taking and autonomy orientations significantly influenced SMEs’ profitability, sales growth, customer satisfaction and new product success.

**Specifications Table**

**Table:** 

Subject area	*Strategic Management, Entrepreneurship*
More specific subject area	*Entrepreneurial Orientation and SME Performance*
Type of data	*Table, figure*
How data was acquired	*Researcher made questionnaire analysis*
Data format	*Raw, analyzed, descriptive and statistical data*
Experimental factors	– Samples consist of owner/managers of SMEs in Lagos State, Nigeria – In this paper, the disposition towards enhancing SME performance through entrepreneurial orientation, especially in a developing economy was studied.
Experimental features	*Entrepreneurial orientation is a critical factor for enhancing SMEs’ performance in developing economies*
Data source location	*SMEs in Lagos State, Nigeria*
Data accessibility	*Data is included in this article*

**Value of the data**

• These data presents demographic attributes of SME owner/managers across industrial sectors in Nigeria, and could be used by other researchers.
• The data describes entrepreneurial orientations and organisational performance of SMEs that could provide insights for other researchers.
• The data allows other researchers to extend the statistical analysis.

## 1 Data

Table 1 represents demographic characteristics of SMEs owners/managers that responded to the research instrument. The data showed that there were more female (55(53.9%)) than male (47(46.1%)). Also, the highest number of respondents were in the age bracket of 26–35 years (54(52.9%)).

**Table 1:** Demographic characteristics of SME owner/Managers.

Parameter	Characteristics	Number (Percentage)
Gender	Male	47 (46.1)
	Female	55 (53.9)
Marital status	Single	68 (66.7)
	Married	34 (33.3)
Age bracket	Under 25years	28 (27.5)
	26–35 years	54 (52.9)
	36–45 years	16 (15.7)
	46 years and Above	4 (3.9)
Years of work experience	Less than 5years	64 (62.7)
	6–10 years	26 (25.5)
	11–15 years	7 (6.9)
	16years and Above	5 (4.9)
Educational qualification	SSCE	9 (8.8)
	HND/Bsc.	72 (70.6)
	MSc./MBA	9 (8.8)
	Others	12 (11.8)
Firm age	1999 and Below	10 (9.8)
	2000 to 2005	19 (18.6)
	2006 to 2011	43 (42.2)
	2012 to Date	30 (29.4)
Nature of business	Agriculture	1 (1.0)
	Manufacturing	10 (9.8)
	Service	91 (89.2)

Table 2 shows the data relationship between entrepreneurship orientation and performance of SMEs. According to the data, proactiveness orientation significantly relates with customer satisfaction (*β* = 0.296, *p* = 0.000) and new product success (*β* = 0.261, *p* = 0.018). Risk-taking orientation also significantly influences profitability (*β* = 0.215, *p* = 0.084), sales growth (*β* = 0.268, *p* = 0.016), new product success (*β* = 0.189, *p* = 0.084). Autonomy orientation had significant effects on profitability (*β* = 0.257, *p* = 0.009), customer satisfaction (*β* = 0.124, *p* = 0.059), new product success (*β* = 0.227, *p* = 0.009). These outcomes closely relate with suggestions from [1], [2], [5].

**Table 2:** Simple multiple regression effects of entrepreneurial orientation on performance of SMEs in Nigeria.

	Profitability		Sales growth		Customer satisfaction		New product success	
Ind variable	β	P-value	β	P-value	β	P-value	β	P-value
Innovativeness	.166	.169	.061	.564	-.025	.758	.066	.530
Proactiveness	-.110	.377	.050	.645	.296	.000***	.261	.018**
Risk taking	.215	.084*	.268	.016***	.055	.507	.189	.084*
Autonomy	.257	.009***	.022	.795	.124	.059**	.227	.009***
Competitive aggressiveness	.086	.441	.071	.472	.054	.466	-.070	.472
R^2^	.171		.126		.235		.209	
Adj R^2^	.128		.080		.195		.168	
F-value	3.962 (5, 06); *p* = 0.003		2.765 (5, 96); *p* = 0.022		5.901 (5, 96); *p* = 0.000		5.068 (5, 96); *p* = 0.000	

* *p* ≤ 0.1,

** *p* ≤ 0.05,

*** *p* ≤0.01

## 2 Experimental design, materials and methods

Survey method has been adopted to gather data. 150 owners/managers of SMEs in three most commercially prominent local governments namely: Ikeja, Lekki and Yaba in Lagos State, Nigeria were included in the research. SMEs are pivotal to this research for their significant contribution to the growth of Nigeria's economy [3], [4], [8]. Questionnaire was used to gather primary data from the respondents. This research benefitted from the ideas of existing research studies. Questions that pertained to entrepreneurial orientation were developed based on [2], while items of firm performance was developed based on [6], [7]. The collated data were coded and entered in SPSS version 22. Data analysis was performed applying descriptive and standard multiple regression statistical test. Ethical consideration in the research process was ensured because administering the questionnaires to respondents was based on their willingness to respond to the research instrument. Moreover, confidentiality and anonymity for participants in the study was assured.
